# Adoptive transfer of immature myeloid cells lacking NF‐κB p50 (p50‐IMC) impedes the growth of MHC‐matched high‐risk neuroblastoma

**DOI:** 10.1002/1878-0261.12904

**Published:** 2021-05-02

**Authors:** Cheng Cui, Theresa Barberi, Rahul Suresh, Alan D. Friedman

**Affiliations:** ^1^ Department of Oncology Johns Hopkins University Baltimore MD USA; ^2^ Department of Physiology China Medical University Shenyang China

**Keywords:** adoptive transfer, immunotherapy, macrophages, myeloid cells, neuroblastoma, NF‐κB p50

## Abstract

High‐risk neuroblastomas harbor abundant myeloid cells that suppress antitumor immunity and support tumor growth. Macrophages lacking the inhibitory NF‐κB p50 subunit adopt a pro‐inflammatory phenotype. We now report that murine 9464D neuroblastoma cells, which express high levels of exogenous *MYCN*, grow slower in syngeneic p50(f/f);Lys‐Cre mice that lack p50 in macrophages and neutrophils, compared with p50(f/f) littermates. Tumors in p50(f/f);Lys‐Cre mice possess increased numbers of total and activated CD4^+^ and CD8^+^ T cells, and depletion of both of these T‐cell populations accelerates tumor growth. Anti‐PD‐1 T‐cell checkpoint blockade, or DNA methyltransferase and histone deacetylase inhibition, further slows tumor growth. In addition, adoptive transfer of immature myeloid cells lacking NF‐κB p50 (p50‐IMC), generated either from the bone marrow of p50^−/−^ mice or via nucleofection of a *p50* sgRNA:Cas9 complex into wild‐type hematopoietic progenitors, also slowed growth of MHC‐matched 9464D tumors but not of MHC‐mismatched Neuro2A tumors. These findings further validate the utility of targeting myeloid NF‐κB p50 as a strategy for cancer therapy and demonstrate activity of p50‐IMC generated by gene editing of syngeneic marrow cells, a cell product relevant to clinical translation.

## Graphical abstract

Myeloid cells lacking NF‐κB p50 adopt a pro‐inflammatory M1 phenotype. Adoptive transfer of p50‐deficient immature myeloid cells (p50‐IMC), generated from either p50‐null mice or via gene editing, impedes the growth of murine neuroblastoma, dependent upon the presence of T cells and MHC‐mediated antigen presentation. Epigenetic therapy or T‐cell checkpoint inhibition cooperates with the absence of myeloid NF‐κB p50 to further slow tumor growth.

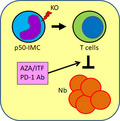

**Abbreviations**
AZA: azacytidine
B6: C57BL/6
DC: dendritic cell
DNMTi: DNA methyltransferase inhibitor
f/f: flox/flox
FC: flow cytometry
HDACi: histone deacetylase inhibitor
HR: highrisk
Lys: lysozyme
*MYCN*‐A: *MYCN*‐amplified
*MYCN*‐NA: *MYCN* nonamplified
Nb: neuroblastoma
p50: NF‐κB p50
TAM: tumor‐associated macrophage
TKI: tyrosine kinase inhibitor
WT: wild‐type

## 1 Introduction

Neuroblastoma (Nb), a malignancy mainly of infants and young children, arises from sympathetic ganglia and the adrenal medulla. Approximately 45% of Nb cases are considered highrisk (HR), especially those over 18 months with metastatic disease beyond regional nodes and patients with *MYCN* gene amplification. Standard treatment for HR Nb cases includes induction chemotherapy, primary tumor resection, autologous stem cell transplantation, radiation therapy, anti‐GD2 antibody with GM‐CSF to mediate antibody‐dependent cellular cytotoxicity, and differentiation therapy using *cis*‐retinoic acid [1]. Approximately 10% of the HR Nb patients enrolled on Children's Oncology Group trial ANBL0532 progressed during induction therapy; among those who did not progress, the 7‐year event‐free survival was ~ 68%, with outcomes worse for the ~ 46% of patients with *MYCN* gene amplification [2]. Therapy for HR Nb also entails significant acute and long‐term side effects, including death from infection, hearing loss, renal dysfunction, secondary cancers, infertility, and growth impairment. Given the limitations of current therapy for HR Nb, additional treatment options are needed.

Multiple solid tumors secrete chemokines and cytokines that attract monocytes and induce their maturation into M2‐polarized tumor‐associated macrophages (TAMs), which suppress T‐cell effector function and secrete growth factors, pro‐angiogenic polypeptides, and pro‐invasive metalloproteinases [3, 4]. In a study focused on Nb with nonamplified *MYCN* (*MYCN*‐NA), M2 TAMs were more abundant in HR patients, those > 18 months with metastatic disease [5]. TAMs induce Nb expression of *MYC* in such cases, leading to a *MYCN*‐amplified (*MYCN*‐A) gene expression signature and worse outcome [6]. A second study found elevated TAMs in three Nb cohorts (including ~ 25% *MYCN*‐A cases) compared with neurofibromas and showed that conversion of Nb TAMs to the pro‐inflammatory M1 phenotype using a CSF1 receptor tyrosine kinase inhibitor (TKI) slows tumor growth in the tyrosine hydroxylase‐*MYCN* transgenic murine HR Nb model [7]. CSF1 receptor TKIs are under clinical investigation; however, resistance to such agents can arise via tumor secretion of alternative M2‐polarizing cytokines [8, 9]. We have therefore been investigating whether targeting the inhibitory NF‐κB p50 (p50) transcription factor in cancer‐associated myeloid cells has therapeutic benefit.

Under basal conditions, NF‐κB p65 (p65) is held in the cytoplasm by IκB, while p50, which has much lower affinity for IκB, is free to enter the nucleus and bind DNA to repress transcription. Pro‐inflammatory cytokines, such as TNF‐α and IL‐1, induce activation of IκB kinase, which then phosphorylates IκB, leading to its degradation by the proteasome. Upon release from IκB, p50:p65 and p65:p65 complexes enter the nucleus, displace p50 dimers, and activate NF‐κB target genes [10, 11]. In myeloid cells lacking p50, pro‐inflammatory genes are de‐repressed, leading to an M1 macrophage phenotype and dendritic cell (DC) activation [12, 13].

Melanoma, fibrosarcoma, colon cancer, glioblastoma, prostate cancer, and pancreatic ductal carcinoma each grow slower in syngeneic B6 p50^−/−^ than in wild‐type (WT) mice, with M2‐to‐M1 TAM reprogramming and increased tumor T‐cell activation evident in p50^−/−^ tumor hosts [14, 15, 16, 17]. In addition, colon cancer grows slower in p50(f/f);Lysozyme(Lys)‐Cre mice that lack p50 only in monocytes, macrophages, and neutrophils [15]. We now report that 9464D Nb cells also grow slower in p50(f/f);Lys‐Cre mice. 9464D cells were derived from transgenic B6 mice that express exogenous *MYCN* from the tyrosine hydroxylase promoter [18, 19]. Nb tumors in p50(f/f);Lys‐Cre hosts have greater numbers of total and activated CD4^+^ and CD8^+^ T cells, and depletion of these T‐cell populations obviates slowed tumor growth. In addition, PD‐1 checkpoint blockade or alternating administration of a DNA methyltransferase inhibitor (DNMTi) and a histone deacetylase inhibitor (HDACi) further slowed tumor growth in p50(f/f);Lys‐Cre mice. Finally, adoptive transfer of immature myeloid cells lacking p50 (p50‐IMC), generated either from the marrow of p50^−/−^ mice or by gene editing the *Nfkb1* alleles encoding p50 in wild‐type marrow myeloid progenitors, also slowed syngeneic 9464D tumor growth. In contrast, p50‐IMC did not slow MHC‐mismatched Neuro2A tumor growth.

These data lend further support to a model in which intrinsically activated, p50‐deficient myeloid cells induce antitumor T‐cell immunity and demonstrate the feasibility of impeding HR Nb tumor growth with adoptively transferred p50‐IMC generated from WT marrow by gene editing.

## 2 Materials and methods

### 2.1 Mice

WT B6 mice were obtained from Charles River Laboratory (Wilmington, MA, USA). p50^−/−^ B6 mice (#6097) were obtained from Jackson Laboratory (Bar Harbor, ME, USA). p50(f/f) and p50(f/f);Lysozyme(Lys)‐Cre B6 mice developed in our laboratory were previously described [16]. Eight‐ to sixteen‐week‐old male and female mice were utilized. This study was carried out in strict accordance with the recommendations in the Guide for the Care and Use of Laboratory Animals of the National Institutes of Health. All efforts were made to minimize suffering. Euthanasia was carried out by carbon dioxide asphyxiation.

### 2.2 Tumor growth analysis

9464D or Neuro2A (ATCC, Manassas, VA, USA) Nb cells were grown in Dulbecco's modified Eagle medium (DMEM) with 10% FBS, 1% nonessential amino acids (NEAA), and 55 µm β‐mercaptoethanol. 1E6 cells in 100 µL PBS were inoculated subcutaneously into the mouse flank, and tumor sizes were monitored two to three times weekly using calipers. Tumor volumes were estimated as length × width × height × π/6. Mice were euthanized when tumors reached 2.0 cm in maximal linear dimension. Anti‐murine PD‐1 antibody (Bio‐X‐Cell, Lebanon, NH, USA; #BE0278) was given intraperitoneally (i.p.) twice weekly at 100 µg per dose for 2 weeks, beginning on day 32. Azacytidine (Sigma, St. Louis, MO, USA; 0.5 mg·kg^−1^ i.p.) and ITF‐2357 (APExBIO, Houston, TX, USA; 2 mg·kg^−1^ i.p.) were given 5 days per week on alternating weeks, beginning on day 33. Anti‐CD4 antibody (Bio‐X‐Cell, BE0003‐1), anti‐CD8 antibody (Bio‐X‐Cell, BE0223), or their combination was given i.p. twice weekly at 100 µg per dose for each antibody beginning on day 21 and continued thereafter.

### 2.3 IMC generation

p50‐IMC were generated from p50^−/−^ mice as described [17]. In brief, marrow mononuclear cells were lineage‐depleted and then expanded in Iscove's modified Dulbecco's medium (IMDM) with 10% heat‐inactivated FBS (HI‐FBS), murine SCF (30 ng·mL^−1^), murine TPO (10 ng·mL^−1^), and murine FL (30 ng·mL^−1^), followed by transfer to IMDM with 10% HI‐FBS and murine M‐CSF (20 ng·mL^−1^) for 24 h on ultra‐low attachment six‐well plates. Nonadherent cells were washed and infused at 1E7 cells/dose in 200 µL PBS via tail vein injection. 5‐fluorouracil (5FU) was given at 150 mg·kg^−1^ i.p. 5 days prior to the first p50‐IMC injection, when tumors were ~ 5 mm in diameter.

To obtain p50KO‐IMC, marrow mononuclear cells from WT mice were lineage‐depleted and placed in serum‐free media (SFM): IMDM, 20% BIT (bovine serum albumin, insulin, transferrin; Stemcell Technologies, Vancouver, BC, Canada), 1% NEAA, 1% sodium pyruvate, SCF, TPO, FL, SR‐1 (750 nm; Cellagen Technology, San Diego, CA, USA), UM171 (35 nm; ExCellThera, Montreal, QC, Canada), and penicillin/streptomycin. Two days later, 2E6 cells were nucleofected with a ribonucleoprotein complex (RNP) consisting of 100 pmol Cas9 and 250 pmol sgRNA (Synthego, Menlo Park, CA, USA; EZ Kit) in 100 µL buffer P3, using a Lonza 4D instrument (Basel, Switzerland) and program DK‐100. The p50 sgRNA sequence, derived from the anti‐sense strand of *Nfkb1* exon 14, is 5′‐U*G*G*AAUGUAAUCCCACCGUA, in which * represents phosphorothioate bonds. As a control, cells were nucleofected with a nontargeting (NT) sgRNA, 5′‐G*C*G*AGGUAUUCGGCUCCGCG. Culture was then continued in SFM between 0.5E6 and 2E6·mL^−1^ until 1 day prior to injection when cells were transferred to IMDM with 20% BIT, 1% NEAA, 1% sodium pyruvate, and M‐CSF. Viable cells were enumerated using a hemocytometer after staining with trypan blue dye.

### 2.4 Tumor myeloid and T‐cell subset and activation analyses

To obtain single‐cell suspensions, Nb tumors were minced and then placed in DMEM with 10% HI‐FBS, Collagenase/Hyaluronidase, Dispase, and DNase I (StemCell, Vancouver, BC, Canada). After incubation at 37 °C for 1 h, cells were passed through a 40‐µm strainer, pelleted, and resuspended at 2E6 viable cells per 100 µL flow cytometry buffer (PBS with 3% HI‐FBS and 5 mm EDTA) on ice. All antibody staining was preceded by 15 min of 1 : 50 FcγR block. Extracellular antibodies were then added and incubated for 45 min. Cells were analyzed using an LSRFortessa Flow Cytometer (BD Biosciences, Menlo Park, CA, USA), gating on cells that excluded Live/Dead Aqua dye (Invitrogen, Carlsbad, CA, USA). Intracellular staining was accomplished using the Foxp3 Staining Kit (Invitrogen). Antibodies were obtained from BD Biosciences unless otherwise indicated. Myeloid subsets were stained with anti‐CD45‐BV650 (BioLegend, San Diego, CA, USA), anti‐CD11b‐FITC, anti‐CD206/MR‐PE‐Cy7, anti‐Ly6G‐BV395 (BioLegend), and anti‐F4/80‐APC (Bio‐Rad, Hercules, CA, USA). To evaluate Tregs, cells were stained with anti‐CD3‐AF488, anti‐CD4‐APC, anti‐CD25‐PerCP‐Cy5.5 (BioLegend), and anti‐Foxp3‐PE. To assess T‐cell activation, total tumor cells were incubated for 4 h at 37 °C in a 5% CO_2_ incubator with Protein Transport Inhibitor Cocktail containing brefeldin A and monensin, or with Cell Stimulation Cocktail containing protein transport inhibitors and PMA/ionomycin (Invitrogen). Cells were then stained with anti‐CD3‐AF488, anti‐CD4‐PE, and anti‐CD8‐BV650 (BioLegend), followed by intracellular stain with anti‐IFN‐γ‐APC (BioLegend) and anti‐TNF‐α‐BV421 (BioLegend). Anti‐PD‐1‐PE‐Cy7 (Invitrogen) was used to analyze PD‐1 expression.

### 2.5 DNA and western blot analyses

Cellular DNA was isolated using the QIAamp DNA Mini Kit (Qiagen, Hilden, Germany). To assess gene‐editing efficiency, a 358 DNA fragment containing the p50 sgRNA cut site near its center was amplified using primers 5′‐AAATACGTGTCAGGGGTGTCC and 5′‐TTCTGCTAACTCTCGGGCAG, followed by Sanger DNA sequencing and then analysis using tide software [20]. Bone marrow‐derived macrophages (BMDM) were generated by culture of marrow cells for 7 days in M‐CSF, as described [16]. Total cellular proteins, prepared in Laemmli sample buffer, were subjected to western blotting using p50 (Cell Signaling, Danvers, MA, USA; 13586), NF‐κB p52 (p52; Cell Signaling; 4881), p65 (Cell Signaling; 8242), and β‐actin (Sigma, AC‐15) antibodies, as described [16]. Band densities were quantified using nih imagej software (imagej.nih.gov).

### 2.6 Data analysis

The Student *t*‐test was used for statistical comparison of tumor volumes at a given time point or tumor T‐cell or myeloid cell subsets. In addition, tumor growth curves were fit to an exponential model using linear mixed‐effects analysis and compared using type II ANOVA statistics [21].

## 3 Results

### 3.1 Absence of myeloid NF‐κB p50 slows high‐risk neuroblastoma tumor growth via T‐cell activation

**Fig. 1:**
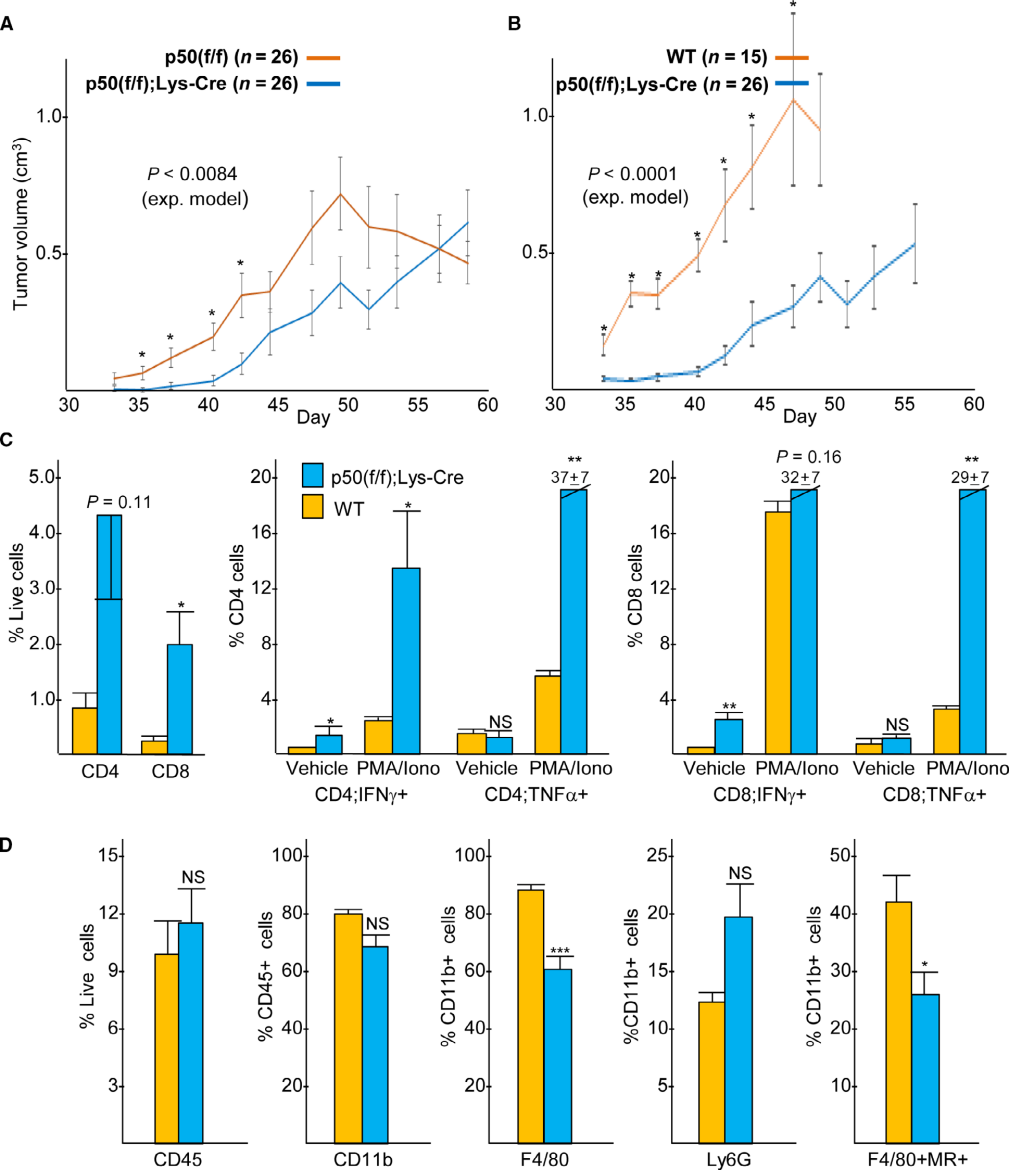
Absence of myeloid NF‐κB p50 slows high‐risk neuroblastoma tumor growth, associated with CD4^+^ and CD8^+^ T‐cell activation. (A, B) 9464D neuroblastoma cells were inoculated into the flanks of syngeneic p50(f/f), p50(f/f);Lys‐Cre, or WT mice. Means and SE for tumor volumes at individual time points are plotted. The *P*‐values shown were calculated using an exponential model to compare the tumor growth curves. In addition, *P* was < 0.05 comparing volumes using Student's *t*‐test on days 35, 37, 40, and 42 for p50(f/f) versus p50(f/f);Lys‐Cre and on days 33 to 47 for WT versus p50(f/f);Lys‐Cre recipients. (C) Tumors isolated on day 46 from WT (*n* = 8) or p50(f/f);Lys‐Cre (*n* = 10) mice were analyzed by flow cytometry for total CD4^+^ and CD8^+^ T cells and for activated T cells, as evidenced by their expression of IFN‐γ or TNF‐α at baseline (vehicle) or after 4 h of stimulation with PMA/ionomycin. Data are combined from two independent experiments (mean values and SEs are shown). (D) Tumors isolated on day 46 from these mice were also analyzed by flow cytometry for the indicated myeloid cell populations among total CD45^+^ hematopoietic tumor cells (mean values and SEs are shown). *P < 0.05; **P < 0.01; ***P < 0.001; NS, not significant.

To determine whether the absence of myeloid NF‐κB p50 slows high‐risk neuroblastoma tumor growth, 9464D cells were inoculated into the flanks of p50(f/f), WT, or p50(f/f);Lys‐Cre mice and tumor volumes were monitored (Fig. 1A,B). Lys‐Cre deletes floxed alleles in granulocytes, activated monocytes, and macrophages [22]. Markedly slower tumor growth was evident in the p50(f/f);Lys‐Cre mice compared with that seen in either p50(f/f) or WT hosts. Survival of tumor‐bearing p50(f/f);Lys‐Cre mice was significantly longer than that of p50(f/f) mice, with survival measured as the time until tumors reached the maximal allowed size (Fig. S1). The apparent decrease in tumor volumes in p50(f/f) and WT hosts at late time points in part reflects the need to euthanize mice with large tumors, obscuring the full magnitude of differential tumor growth between control mice and p50(f/f);Lys‐Cre mice at later time points. The reason for the evident more rapid tumor growth in WT compared with p50(f/f) mice is uncertain but could reflect a mild effect of the *lox*P sites surrounding *Nfkb1* exon 4 on p50 expression.

On day 46, total and activated CD4^+^ and CD8^+^ T cells within intermediate‐sized tumors in WT or p50(f/f);Lys‐Cre mice were analyzed by flow cytometry (Fig. 1C). WT mice were used for this comparison to study the full effect of *Nfkb1* exon 4 deletion. CD4^+^ T‐cell numbers were increased fivefold (*P* = 0.11), and CD8^+^ T‐cell numbers were increased ~ 8‐fold (*P* < 0.05), in p50(f/f);Lys‐Cre compared with WT mice. Under basal conditions, a significantly greater proportion of CD4^+^ and CD8^+^ T cells from the tumors of p50(f/f);Lys‐Cre mice expressed IFN‐γ compared with T cells from control mice. Upon *in vitro* PMA/ionomycin stimulation, IFN‐γ and TNF‐α expression markedly increased in CD4^+^ T cells from tumors in p50(f/f);Lys‐Cre mice, and the percentage of CD8^+^ T cells expressing IFN‐γ was also increased. The proportion of Foxp3^+^CD25^+^ regulatory T cells (Tregs) among CD4^+^ T cells was nearly identical in tumors developing in p50(f/f);Lys‐Cre mice compared with WT tumor mice (Fig. S2A).

Myeloid cells within these tumors were also evaluated (Fig. 1D). CD45 is a pan‐hematopoietic surface protein, and CD11b is a pan‐myeloid marker. The proportion of CD45^+^ and CD11b^+^ cells within tumors developing in WT or p50(f/f);Lys‐Cre mice did not differ. The absence of p50 led to a modest, 1.5‐fold, reduction in total F4/80^+^ macrophages and to a 1.7‐fold, but variable, increase in Ly6G^+^ neutrophils. Mannose receptor (MR, CD206) is a marker of M2‐polarized macrophages. The proportion of MR^+^ tumor macrophages was reduced almost twofold in p50(/f/);Lys‐Cre mice, consistent with the known role of p50 in promoting macrophage M2 polarization. Western blot analysis of BMDM derived from p50(f/f) and p50(f/f);Lys‐Cre mice confirmed our prior finding of reduced p50 protein [16], and in addition demonstrates mild reduction in the related p52 NF‐κB subunit and decreased p65 (Fig. S3). Splenic B cells from p50‐null mice also have reduced p52 and p65 protein levels [23].

**Fig. 2:**
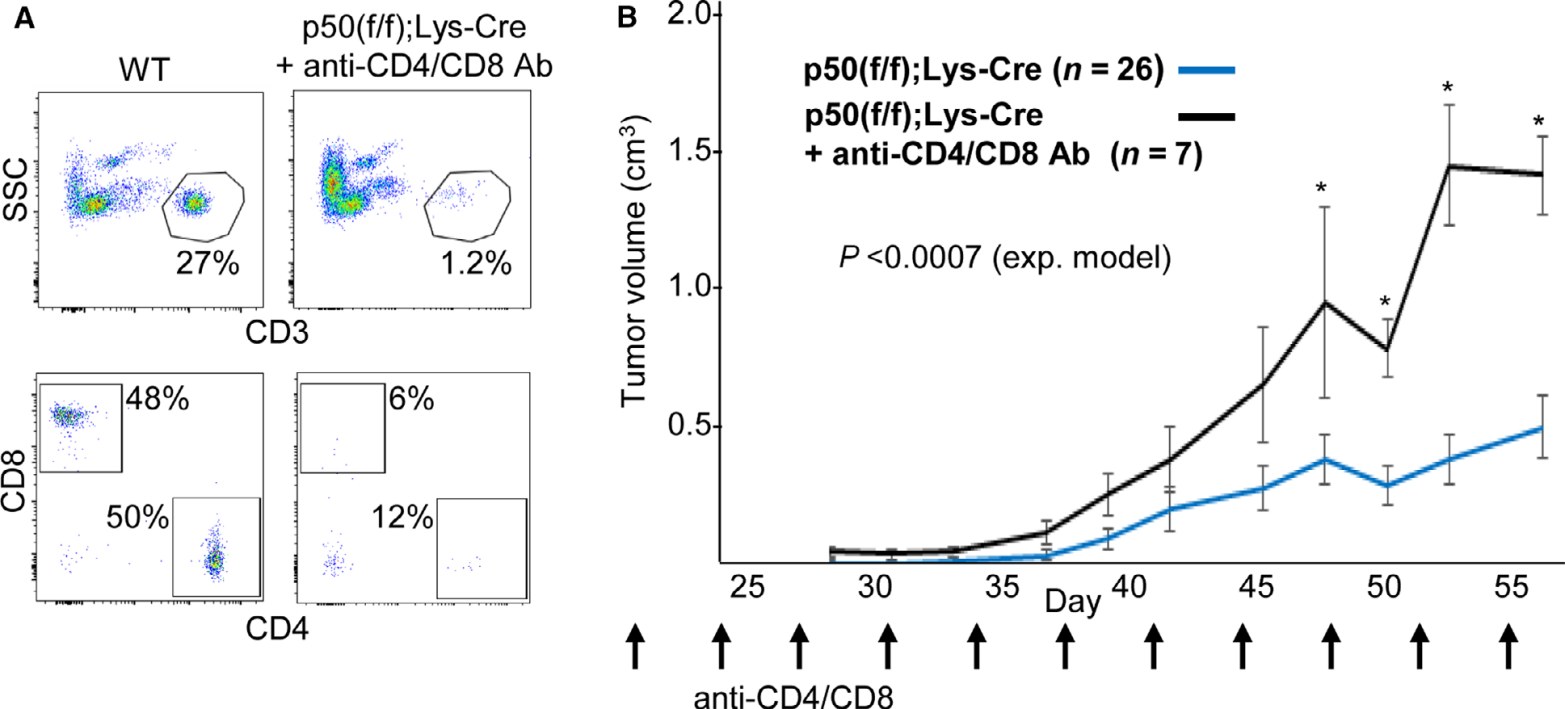
Depletion of CD4^+^ and CD8^+^ T cells accelerates tumor growth in p50(f/f);Lys‐Cre mice. (A) Peripheral blood cells from a WT mouse and from a p50(f/f);Lys‐Cre mouse treated with anti‐CD4 and anti‐CD8 antibodies twice weekly from day 21 to day 55 after 9464D tumor cell inoculation were subjected to flow cytometry for CD3, CD4, and CD8. The percent CD3^+^ cells among nucleated blood cells and the percent CD4^+^ or CD8^+^ cells among CD3^+^ cells are shown for each sample. (B) 9464D cells were inoculated into the flanks of p50(f/f);Lys‐Cre mice, which then received either no treatment (data from Fig. 1A) or anti‐CD4 and anti‐CD8 antibody (100 µg each) twice weekly starting on day 21. Tumor volumes (mean values and SEs) are shown at individual time points. The *P*‐value shown compares the entire growth curves using an exponential model. In addition, *P* was < 0.05 comparing volumes on days 49 to 56.

Having seen slowed tumor growth and increased total and activated CD4^+^ and CD8^+^ T cells in tumors in p50(f/f);Lys‐Cre mice, we sought to determine whether depletion of CD4^+^ or CD8^+^ T cells would accelerate tumor growth. Twice weekly injections of anti‐CD4 and anti‐CD8 antibody effectively reduced blood CD4^+^ and CD8^+^ T‐cell numbers (Fig. 2A). When both T‐cell subsets were depleted beginning 21 days after tumor cell inoculation, we observed markedly increased tumor growth (Fig. 2B), with tumor volumes increasing at a rate similar to that seen in p50(f/f) mice (Fig. S4A). Notably, depletion of CD4^+^ T cells alone also increased the rate of tumor growth, but to a lesser extent (Fig. S4B), depletion of CD8^+^ T cells alone did not accelerate tumor growth (Fig. S4C), indicating that both CD4^+^ and CD8^+^ T cells make important contributions to slowed Nb tumor growth in p50(f/f);Lys‐Cre mice. Overall, these data support a model in which the absence of tumor myeloid cell NF‐κB p50 leads to the activation of tumor CD4^+^ and CD8^+^ T cells to slow 9464D high‐risk neuroblastoma tumor growth.

### 3.2 T‐cell checkpoint inhibition cooperates with the absence of myeloid NF‐κB p50 to slow high‐risk neuroblastoma tumor growth

**Fig. 3:**
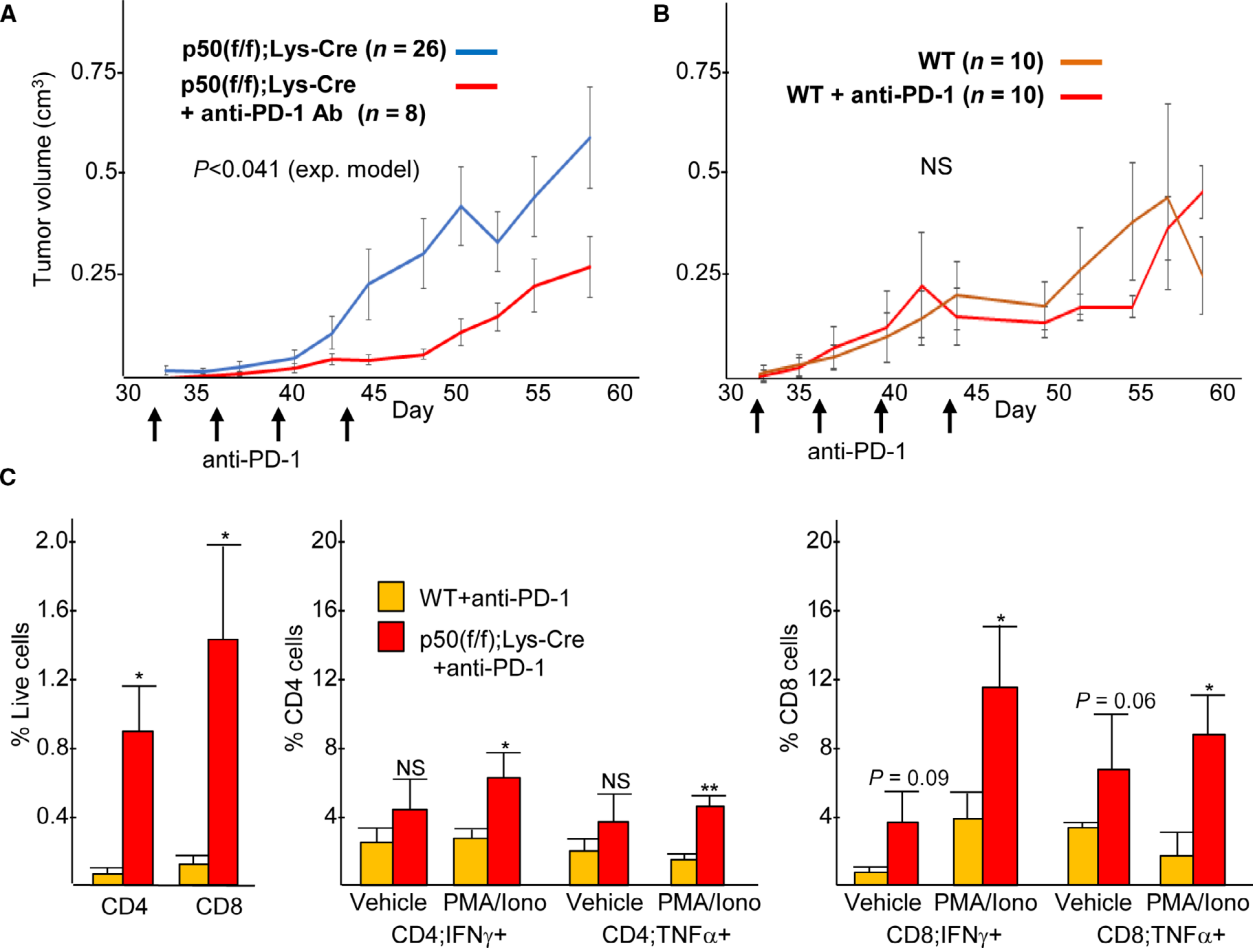
T‐cell checkpoint inhibition cooperates with the absence of myeloid NF‐κB p50 to slow high‐risk neuroblastoma tumor growth. (A, B) 9464D cells were inoculated into the flanks of p50(f/f);Lys‐Cre mice or WT mice, which then received either no treatment or anti‐PD‐1 antibody (100 µg) twice weekly for 2 weeks starting on day 32. Tumor volumes (mean values and SEs) are shown at individual time points. The *P*‐value shown compares the entire growth curves using an exponential model. *P* was not < 0.05 on any individual day. p50(f/f);Lys‐Cre no treatment data are the same as that shown in Fig. 1A. WT no treatment data were obtained in the same experiment as the WT+ anti‐PD‐1 treatment group. (C) Tumors isolated on day 46 were analyzed by flow cytometry for total and activated CD4^+^ and CD8^+^ T cells, the latter at baseline (vehicle) or after 4 h of stimulation with PMA/ionomycin (*n* = 5, mean values and SEs are shown).

The PD‐1 T‐cell checkpoint protein was detected on the surface 43% of CD4^+^ T cells and 77% of CD8^+^ T cells isolated from 9464D Nb tumors growing in p50(f/f);Lys‐Cre hosts and to a similar extent in tumor from p50(f/f) mice (Fig. S5A). To determine whether PD‐1 inhibition cooperates with the absence of myeloid NF‐κB p50 to slow Nb tumor growth, p50(f/f);Lys‐Cre mice were inoculated with 9464D cells and then received four injections of anti‐PD‐1 antibody between days 32 and 43. PD‐1 blockade significantly slowed Nb tumor growth beyond that of untreated p50(f/f);Lys‐Cre mice (Fig. 3A). In contrast, PD‐1 antibody did not impact tumor growth in WT or p50(f/f) mice (Fig. 3B and Fig. S5B). Untreated WT mice analyzed for this experiment were studied concurrently with those treated with PD‐1 antibody and are separate from those presented in Fig. 1B; the reason for slower tumor growth in untreated mice in this experiment is uncertain.

Tumor CD4^+^ and CD8^+^ T cells in PD‐1 antibody‐treated WT or p50(f/f);Lys‐Cre mice were examined by flow cytometry (Fig. 3C). Upon PD‐1 blockade, tumors in p50(f/f);Lys‐Cre mice exhibited a marked increase in both CD4^+^ and CD8^+^ T‐cell numbers, above that of WT mice that also received PD‐1 antibody. Of note, the proportion of CD4^+^ and CD8^+^ T cells among live cells was reduced in this experiment compared with that presented in Fig. 1C, perhaps reflecting differences in tumor cell viability. Additionally, the fraction of CD4^+^ and CD8^+^ T cells expressing IFN‐γ and TNF‐α was increased after PMA/ionomycin stimulation. The number of Tregs was significantly higher in the tumors from PD‐1 antibody‐treated p50(f/f);Lys‐Cre mice compared with WT mice (Fig. S2B). Together, these data indicate that the absence of myeloid NF‐κB p50 augments the activity of T‐cell checkpoint blockade to increase T‐cell infiltration or expansion, as well as T‐cell activation, to slow high‐risk neuroblastoma tumor growth.

### 3.3 Epigenetic therapy cooperates with the absence of myeloid NF‐κB p50 to slow high‐risk neuroblastoma tumor growth

**Fig. 4:**
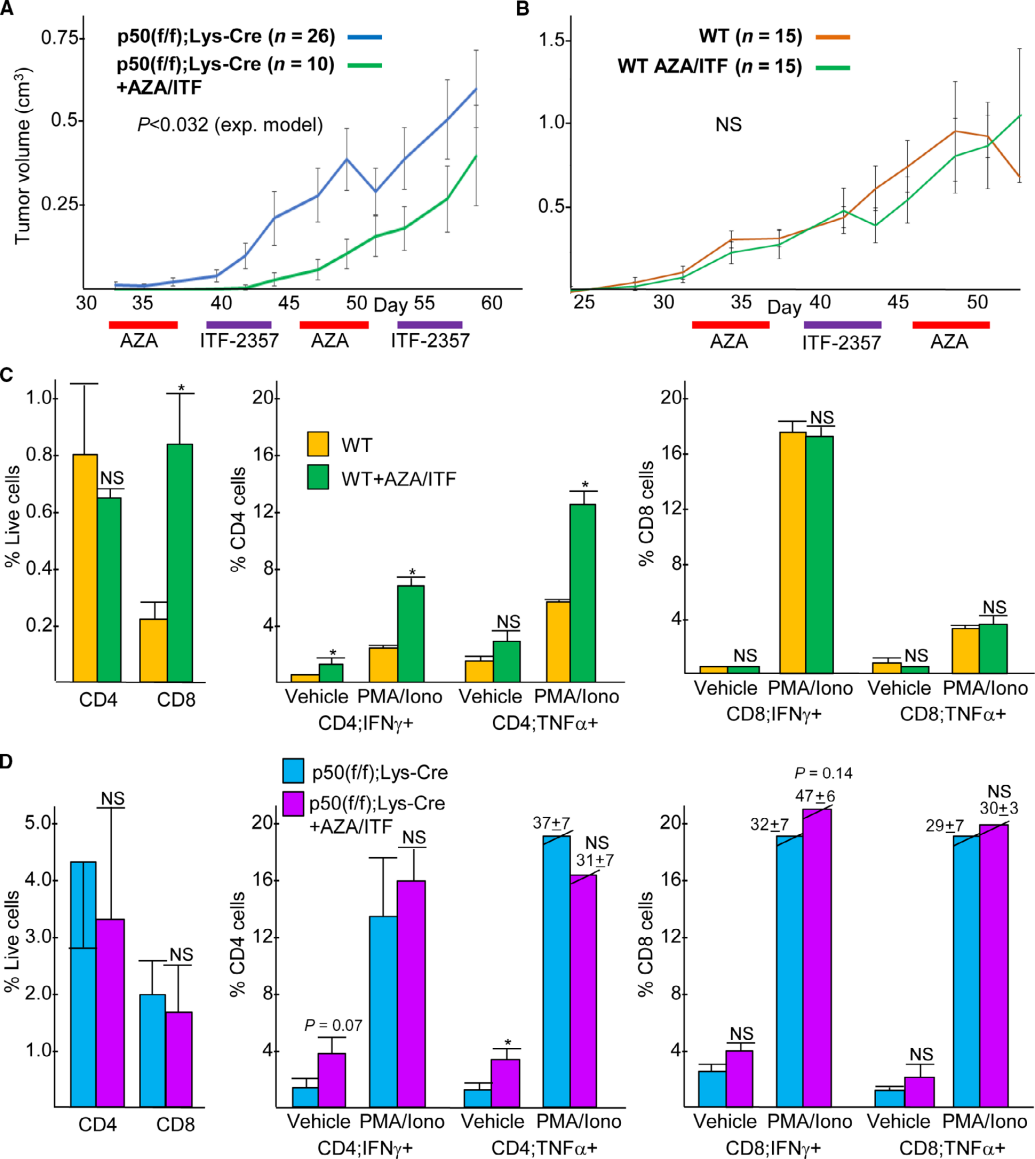
DNA methyltransferase plus histone deacetylase inhibition cooperates with the absence of myeloid NF‐κB p50 to slow high‐risk neuroblastoma tumor growth. (A, B) 9464D cells were inoculated into the flanks of p50(f/f);Lys‐Cre mice or WT mice, which then received either no treatment or azacytidine alternating with ITF‐2357 weekly starting on day 33. Tumor volumes (mean values and SEs) are shown at individual time points. The *P*‐values shown compare the entire growth curves using an exponential model. *P* was not < 0.05 on any individual day (*P* = 0.055 on day 49). p50(f/f);Lys‐Cre no treatment data are the same as that shown in Fig. 1A. (C, D) Tumors isolated on day 46 were analyzed by flow cytometry for total and activated CD4^+^ and CD8^+^ T cells, the latter at baseline or after 4 h of stimulation with PMA/ionomycin (*n* = 8 for WT and for p50(f/f);Lys‐Cre, *n* = 10 for WT+AZA/ITF and for p50(f/f);Lys‐Cre+AZA/ITF; mean values and SEs are shown).

Sequential low‐dose DNMT and HDAC inhibition has been demonstrated to slow tumor growth, at least in part via induction of endogenous retroviruses and the resulting production of IFN‐α/β [24]. IFN‐α/β may activate tumor myeloid cells and T cells directly and by inducing tumor cell secretion of additional pro‐inflammatory cytokines. To determine whether sequential DNMTi and HDACi cooperate with the absence of myeloid NF‐κB p50 to slow Nb tumor growth, p50(f/f);Lys‐Cre mice were inoculated with 9464D cells and then on day 33 began to receive azacytidine for 5 days followed by ITF‐2357 (Givinostat) for 5 days, on alternating weeks until the end of the study. These agents cooperated with the absence of myeloid p50 to significantly slow tumor growth beyond that of untreated mice (Fig. 4A). In contrast, azacytidine combined with ITF‐2357 did not slow tumor growth in WT tumor mice (Fig. 4B), and azacytidine alone given biweekly did not slow tumor growth in p50(f/f);Lys‐Cre mice (Fig. S6).

In WT mice, epigenetic therapy increased the number of tumor CD8^+^ T cells and increased the proportion of CD4^+^ T cells expressing IFN‐γ and TNF‐α (Fig. 4C). In contrast, in p50(f/f);Lys‐Cre mice, azacytidine and ITF‐2357 did not augment T‐cell numbers or activation beyond the increases already evident due to the absence of myeloid NF‐κB p50, with the exception of an increased proportion of CD4^+^TNF‐α^+^ T cells in vehicle (Fig. 4D). In addition, azacytidine and ITF‐2357 did not impact the proportion of tumor Tregs, CD11b^+^ myeloid cells, F4/80^+^ macrophages, Ly6G^+^ neutrophils, or MR^+^ macrophages in tumors from WT or p50(f/f);Lys‐Cre Nb mice (Figs S2C and S7). Thus, while low‐dose DNMTi and HDACi cooperate with the absence of myeloid NF‐κB p50 to slow HR neuroblastoma tumor growth, we did not identify any alterations in the number or phenotype of tumor T cells or myeloid cells to account for the benefit of azacytidine and ITF‐2357 in this setting.

### 3.4 Adoptive transfer of p50‐IMC generated from p50‐null mice slows MHC‐matched high‐risk neuroblastoma tumor growth

**Fig. 5:**
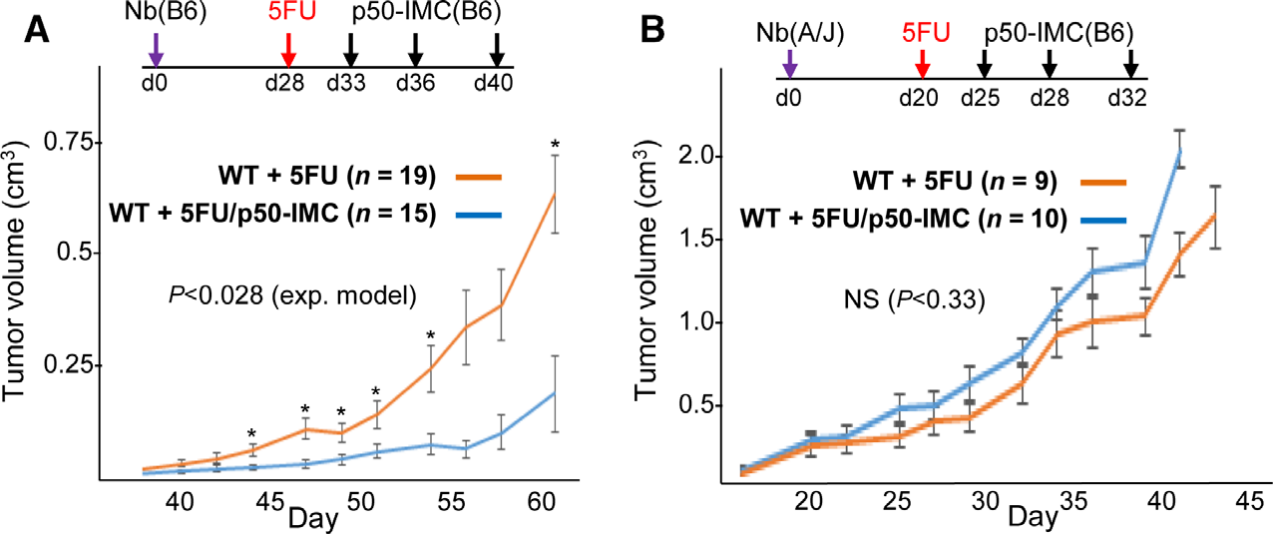
Effect of p50‐IMC on MHC‐matched and MHC‐mismatched neuroblastoma tumor growth. (A) WT B6 mice inoculated with B6‐derived 9464D Nb cells on day 0 received 5FU on day 28, followed either by no additional therapy or by 1E7 B6‐derived p50‐IMC on days 33, 36, and 40. The *P*‐value shown compares the entire growth curves using an exponential model. *P* was < 0.05 based on Student's *t*‐test comparing tumor volumes on days 44, 47, 49, 51, 54, and 61. (B) WT A/J mice inoculated with A/J‐derived Neuro2A Nb cells on d0 received 5FU on day 20, followed either by no additional therapy or by 1E7 B6‐derived p50‐IMC on days 25, 28, and 32.

We previously demonstrated that adoptive transfer of p50‐IMC, following a dose of myelodepleting 5FU, slows syngeneic murine prostate cancer and pancreatic ductal carcinoma tumor growth [17]. B6 mice inoculated subcutaneously with syngeneic 9464D Nb cells received a dose of 5FU on day 28, when tumors were ~ 5 mm in diameter, and were then left untreated or received three doses of 1E7 B6‐derived p50‐IMC, as diagrammed (Fig. 5A, top). As done previously, p50‐IMC were generated by expansion of myeloid progenitors from the marrow of p50^−/−^ mice using cytokines that maintain myeloid progenitor immaturity, followed by transfer to M‐CSF for 1 day prior to injection. Administration of p50‐IMC markedly slowed 9464D Nb tumor growth (Fig. 5A). In contrast, B6‐derived p50‐IMC (H‐2K^b^) did not significantly slow the growth of MHC‐mismatched A/J‐derived Neuro2A Nb tumors (H‐2K^k^) in A/J mice, further supporting a model in which p50‐IMC impair tumor growth predominantly via T‐cell activation (Fig. 5B).

### 3.5 Adoptive transfer of p50KO‐IMC generated be gene editing slows high‐risk neuroblastoma tumor growth

**Fig. 6:**
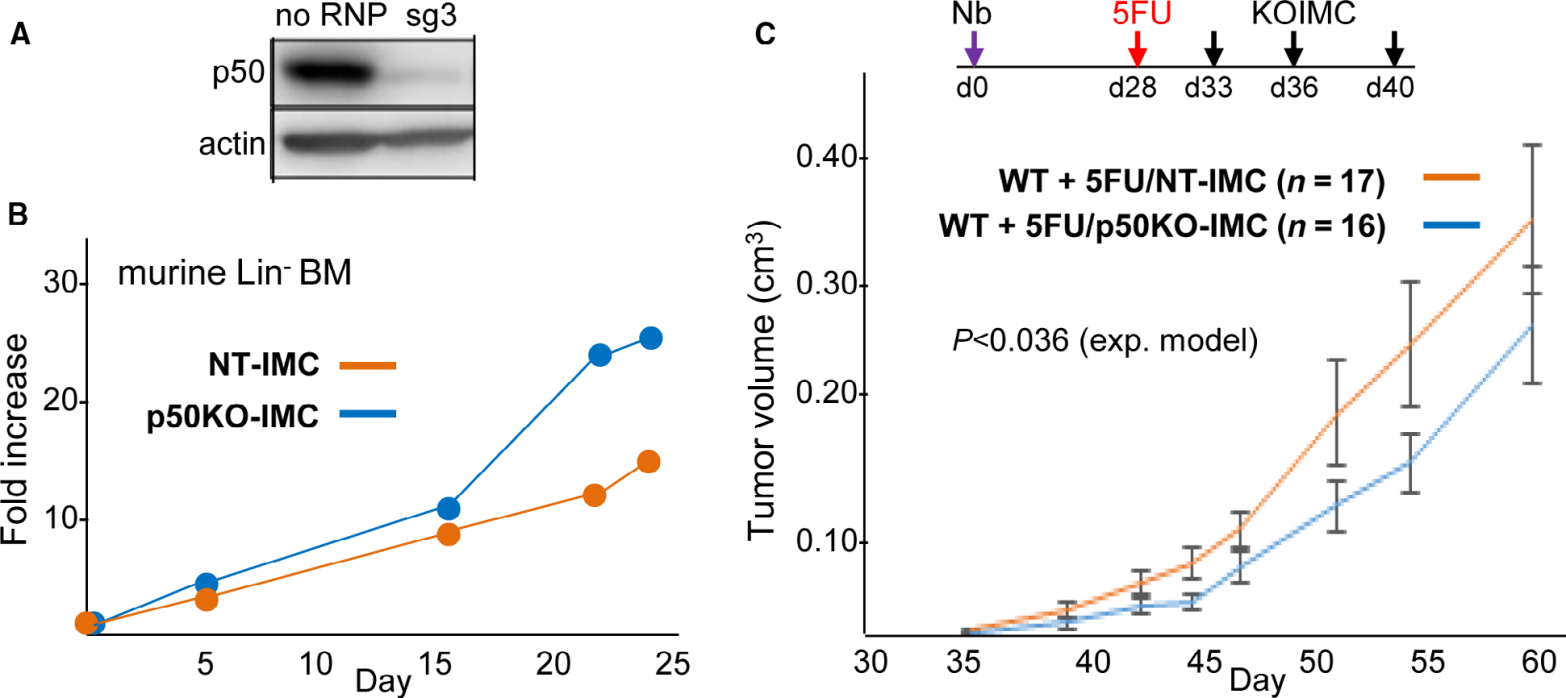
p50‐KOIMC slows high‐risk neuroblastoma tumor growth. (A) Lineage‐negative (Lin^−^) murine marrow cells were nucleofected with sg3 (targeting exon 14 of *Nfkb1*) complexed with Cas9, or with no ribonucleoproteins (RNPs). Western blotting for NF‐κB p50 and β‐actin was performed 18 days later using total cellular protein from 2E5 cells. (B) 2E6 Lin^−^ murine marrow cells were nucleofected with Cas9:p50 sg3 RNP or a nontargeting (NT) RNP and then cultured in IMDM with serum‐free BIT, SCF, TPO, FL, SR‐1, and UM171. The fold change in viable cell numbers, with data shown representative of three independent experiments. (C) WT mice inoculated with Nb cells on day 0 received 5FU on day 28, followed by 1E7 nontargeting NT‐IMC or *Nfkb1* gene‐edited p50KO‐IMC on days 33, 36, and 40. The *P*‐value shown compares the entire growth curves using an exponential model. *P* was 0.031 comparing tumor volumes on day 44.

As one path toward clinical translation, we envision using CRISPR/Cas9 to knock out (KO) the alleles encoding p50 in patient‐derived myeloid progenitors followed by their expansion *ex vivo* and reinfusion after a dose of myelodepleting chemotherapy. Lentiviral‐mediated gene editing carries the risk that the virus will activate oncogenes in the vicinity of vector insertion sites, and ongoing sgRNA and Cas9 expression increases the chances for off‐target mutations. We therefore targeted the *Nfkb1* gene encoding p50 by nucleofecting cells with ribonucleoprotein (RNP) complexes comprised of Cas9 protein and an *Nfkb1*‐specific sgRNA. After screening several sgRNAs, we selected the one (sg3) that most effectively reduced p50 protein expression and used it to generate p50KO‐IMC by nucleofecting lineage‐depleted murine marrow cells in serum‐free media to minimize exposure to nucleases and proteases (Fig. 6A). PCR amplification of edited and control DNA, followed by DNA sequencing and TIDE analysis, confirmed highly efficient *Nfkb1* exon 14 gene editing, with a single base pair deletion predominating (Fig. S8A). Potential off‐target edits predicted by Cas‐OFFinder [25] are shown, allowing 0–3 mismatches and one DNA or RNA bulge (Fig. S8B); there are no exact off‐target matches, and no potential sites with one or two mismatches are found in exons. Control cells were nucleofected with a nontargeting sgRNA to generate NT‐IMC. As the use of serum‐free media is desirable during clinical translation, we further expanded gene‐edited murine marrow progenitors in serum‐free BIT with SCF, TPO, and FL, cytokines that maintain stem/progenitor cell immaturity, together with SR‐1 and UM171, small molecules that enhance CD34^+^ hematopoietic stem/progenitor cell expansion [26, 27]. Gene‐edited cells expanded 25‐ to 30‐fold over a 24‐day period, with p50KO‐IMC reproducibly expanding to a 1.5‐fold greater extent than control NT‐IMC (Fig. 6B). B6 mice inoculated with 9464D Nb cells received a dose of 5FU on day 28 followed by three doses of either NT‐IMC or p50KO‐IMC, with infusion of p50KO‐IMC leading to significantly slower neuroblastoma tumor growth (Fig. 6C).

## 4 Discussion

Prior work demonstrating slowed tumor growth in the absence of host NF‐κB p50 largely utilized p50^−/−^ mice, with only one study showing slowed colon cancer tumor progression in mice lacking p50 specifically in activated monocytes, macrophages, and neutrophils [15]. We now find that *MYCN*‐A high‐risk neuroblastoma tumor growth is also reduced in p50(f/f);Lys‐Cre mice, further validating the idea of targeting myeloid p50 as a therapeutic strategy for cancer therapy.

Moreover, our study is the first to investigate the role of tumor T‐cell numbers and activation in the setting of myeloid‐specific p50 deletion. Depletion of both CD4^+^ and CD8^+^ T cells eliminated the effect of global absence of p50 on colon cancer tumor growth. In p50^−/−^ mice injected intracranially with syngeneic glioblastoma cells, CD4^+^ T‐cell depletion completely eliminated slowed tumor growth, whereas CD8^+^ T‐cell depletion only partially obviated the effect [15, 16]. However, naïve p50^−/−^ CD8^+^ T cells manifest increased activation compared with WT CD8^+^ T cells when placed in Tc1‐polarizing conditions, potentially accounting, in part, for the reduction in tumor growth in p50^−/−^ mice [16]. We now find that Nb tumors developing in p50(f/f);Lys‐Cre mice have increased total and activated CD4^+^ and CD8^+^ T cells and that the combined depletion of both of these T‐cell subsets leads to tumor growth at a rate similar to that seen in control mice. These data substantially strengthen the concept that the absence of myeloid NF‐κB p50 slows tumor growth by increasing tumor T‐cell numbers and/or activation.

B lymphocytes from p50^−/−^ mice have reduced levels of p52, p65, c‐Rel, and RelB [23]. Consistent with these data, we find that BMDM derived from p50(f/f);Lys‐Cre mice not only have reduced p50, but also have reduced p52 and p65 compared with BMDM generated from p50(f/f) mice. Thus, the phenotypes of p50‐deficient myeloid cells, including their ability to activate T cells to slow tumor growth, may in part reflect deficiencies in additional NF‐κB subunits.

Both PD‐1 T‐cell checkpoint blockade and low‐dose alternating DNMTi and HDACi further slowed Nb tumor growth in p50(f/f);Lys‐Cre mice. Anti‐PD‐1 antibody treatment increased tumor T‐cell numbers and activation in this setting, likely by activating the 40% of tumor CD4^+^ T cells and 70% of tumor CD8^+^ T cells expressing PD‐1, a marker of T‐cell exhaustion. Azacytidine alternated with ITF‐2357 increases IFN‐α/β pathway activation via induction of endogenous retroviruses in non‐small cell lung cancer, potentially leading to expression of cytokines that activate tumor myeloid and T cells; in addition, this epigenetic therapy downmodulates lung cancer *MYC* mRNA expression to contribute to slowed tumor growth [24]. Perhaps a similar effect on Nb *MYC* expression, or effects on additional genes that directly impact Nb cell proliferation, enables azacytidine and ITF‐2357 to slow Nb tumor growth in p50(f/f);Lys‐Cre mice even though these agents did not further augment Nb tumor T‐cell activation; we will investigate this possibility in the future. Of note, in the lung cancer model, 12 weeks of exposure to these epigenetic modulators increased the number of activated, CD8^+^IFN‐γ^+^ tumor T cells, but this change was not evident at 4 weeks [24], which was the length of time we exposed mice to these agents to observe a significant difference in tumor growth. In addition, both HDACi and DNMTi augment Nb differentiation, which might also allow these agents to cooperate with the absence of myeloid p50 to slow Nb tumor growth [28, 29]. HDACi interference with transcriptional repression mediated by the small amount of p50 remaining in mature myeloid cells present in p50(f/f);Lys‐Cre mice may also contribute to slowed tumor growth [11].

While a small molecule that targets NF‐κB p50 might be developed, this could prove challenging due to the lack of a high‐affinity pocket and homology to NF‐κB p65. In addition, such a molecule would also activate NF‐κB in malignant or premalignant cells, which might prove detrimental; for example, *Nfkb1* ^−/−^ mice exposed to a chemical mutagen have accelerated development of hepatocellular carcinoma [30]. On the other hand, a subset of high‐risk neuroblastomas harbor increased expression of BARD1, a protein that complexes with BRCA1 to maintain genomic stability but also has oncogenic properties [31]. BARD1 directly interacts with and stabilizes NF‐κB p50 [32], which might favor transformation, including via p50‐dependent induction of *Bcl2* gene expression [33, 34].

We have pursued a therapeutic strategy utilizing adoptive transfer of immature myeloid cells lacking p50 (p50‐IMC). The use of immature cells increases tumor localization and minimizes adherence to solid organs including liver, a problem seen in clinical trials when mature macrophages are infused [35]. p50‐IMC develop into both TAMs and DCs *in vivo*, and IMC localize strongly to prostate cancer and pancreatic ductal carcinoma tumors, with tumor localization ~ 2.5‐fold higher than spleen or lung with minimal liver localization [17]. We previously found that a dose 5FU followed by three infusions of p50‐IMC slows the growth of syngeneic murine prostate cancer and pancreatic ductal carcinoma, whereas neither 5FU nor p50‐IMC alone were effective [17]. 5FU induces a nadir of host white blood cells 4 days after administration, analogous to lymphodepletion prior to adoptive T‐cell transfer in patients. In addition, 5FU targets immunosuppressive tumor myeloid cells [36] and may release tumor neoantigens to further enhance antitumor response.

We now find that 5FU followed by three doses of p50‐IMC generated from p50^−/−^ mice also markedly slows the growth of syngeneic murine high‐risk 9464D Nb, extending the potential utility of this immunotherapy approach. In contrast, B6‐derived p50‐IMC did not slow the growth of MHC‐mismatched Neuro2A Nb, indicating the MHC‐dependent rather than nonspecific T‐cell activation due to cytokine secretion is central to the efficacy of p50‐IMC. In addition, we find for the first time that 5FU followed by three doses of p50KO‐IMC, generated by gene editing the *Nfkb1* alleles in WT marrow and then expanding cells in serum‐free media, is also effective at slowing tumor growth. In the future, we will endeavor to further optimize efficacy of this clinically relevant cell therapy in immune‐competent murine models, alone and in combination with additional agents or cell modifications, while also developing human p50KO‐IMC for evaluation in patients.

## Conflict of interest

ADF is a named inventor on a PCT patent application entitled ‘NF‐κB p50 Deficient Immature Myeloid Cells and Their Use in Treatment of Cancer’ filed by the Johns Hopkins University.

## Author contributions

CC, TB, RS, and ADF conceived, designed, and performed experiments, and undertook statistical analysis and manuscript writing or editing.

## Supporting information


**Fig. S1.** Tumor growth data for 9464D Nb cells inoculated into p50(f/f) or p50(f/f);Lys‐Cre, as presented in Figure 1A, were further analyzed by plotting murine survival versus days after tumor inoculation.

**Fig. S2.** Analysis of high‐risk neuroblastoma tumors for regulatory T cells.

**Fig. S3.** Expression of NF‐κB p50, p52, and p65 in BMDM derived from p50(f/f) = ff and p50(f/f);Lys‐Cre = ff;Cre mice.

**Fig. S4.** Depletion of CD4^+^ and CD8^+^ T cells, and to a lesser extent CD4^+^ T cells alone, accelerates tumor growth in p50(f/f);Lys‐Cre mice.

**Fig. S5.** T cell checkpoint inhibition does not slow high‐risk neuroblastoma tumor growth in p50(f/f) mice.

**Fig. S6.** 9464D cells were inoculated into the flanks of p50(f/f);Lys‐Cre mice, which then received either no treatment or azacytidine biweekly starting on day 33.

**Fig. S7.** Analysis of high‐risk neuroblastoma tumors exposed to AZA/ITF‐2357 for myeloid cell subsets.

**Fig. S8.** Analysis of insertions and deletions generated by *Nfkb1* gene editing.

## Data Availability

The raw data are available from the corresponding author upon reasonable request.
