# Supplementary figures and images for: Adoptive transfer of immature myeloid cells lacking NF‐κB p50 (p50‐IMC) impedes the growth of MHC‐matched high‐risk neuroblastoma
Source: Mol Oncol. 2021 May 2;15(7):1783–96. doi: 10.1002/1878-0261.12904 (PMC8253086; doi:10.1002/1878-0261.12904)

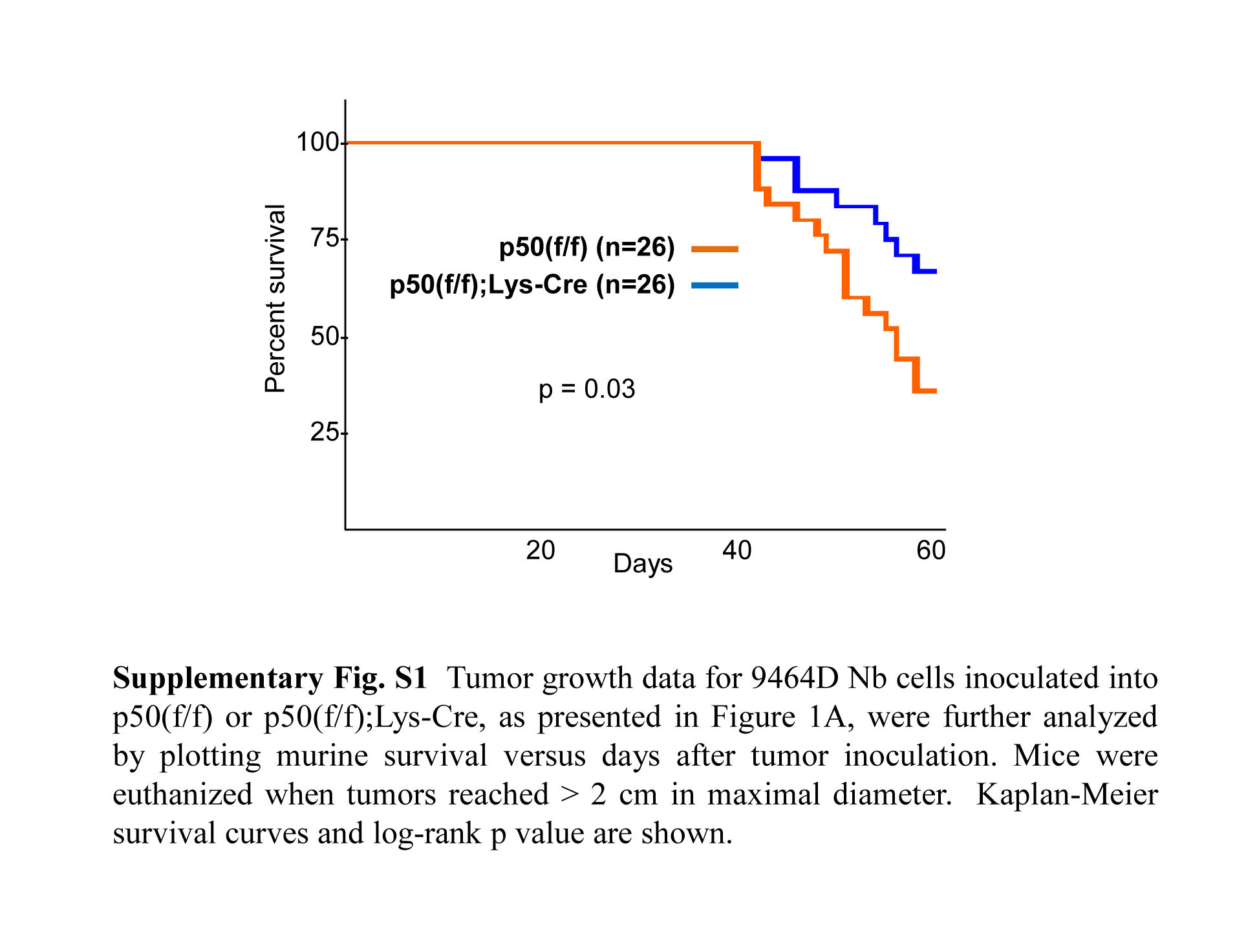

Supplement: Supplementary file 1 — Fig. S1. Tumor growth data for 9464D Nb cells inoculated into p50(f/f) or p50(f/f);Lys‐Cre, as presented in Figure 1A, were further analyzed by plotting murine survival versus days after tumor inoculation. [file MOL2-15-1783-s007.tif]

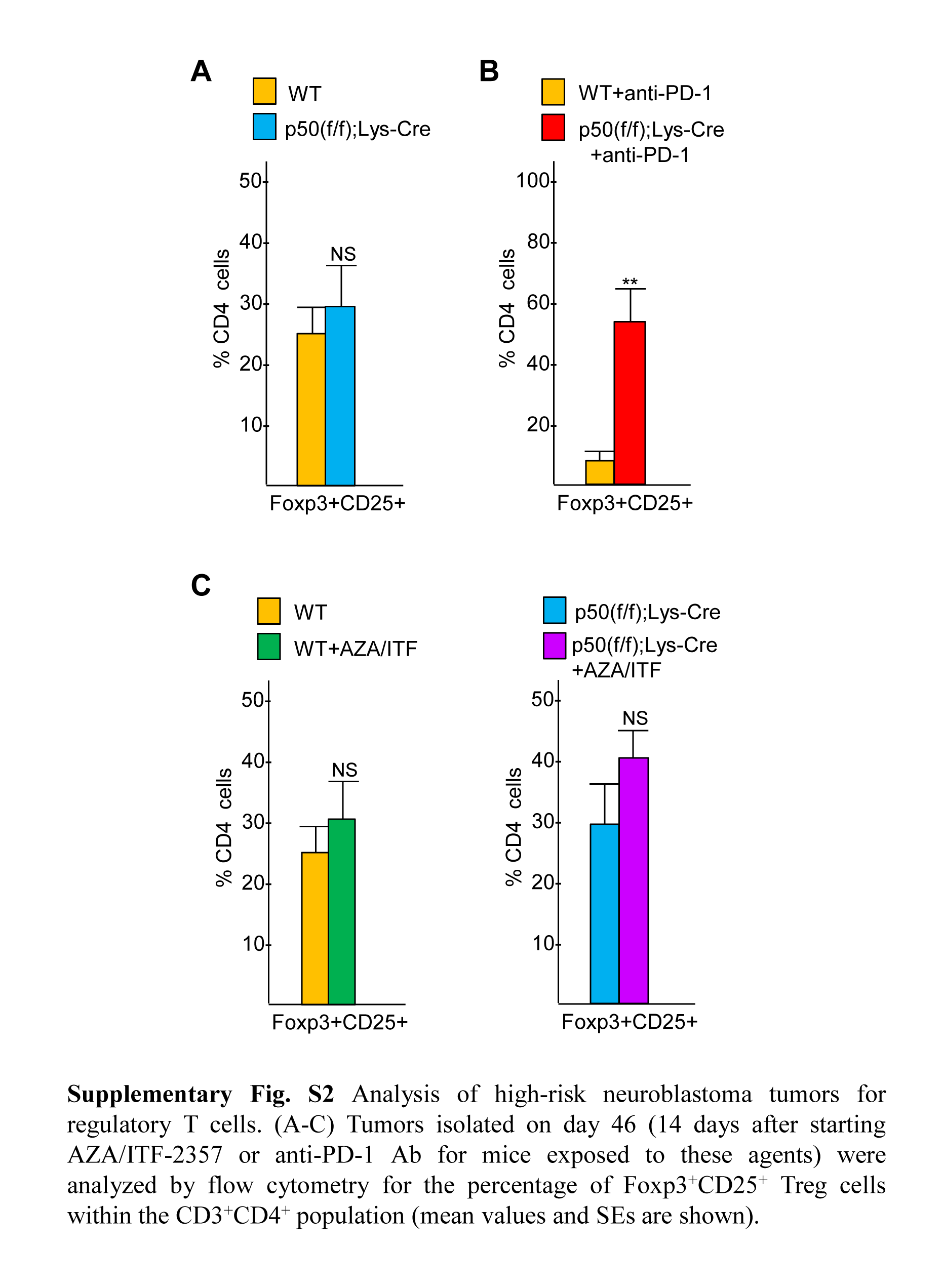

Supplement: Supplementary file 2 — Fig. S2. Analysis of high‐risk neuroblastoma tumors for regulatory T cells. [file MOL2-15-1783-s004.tif]

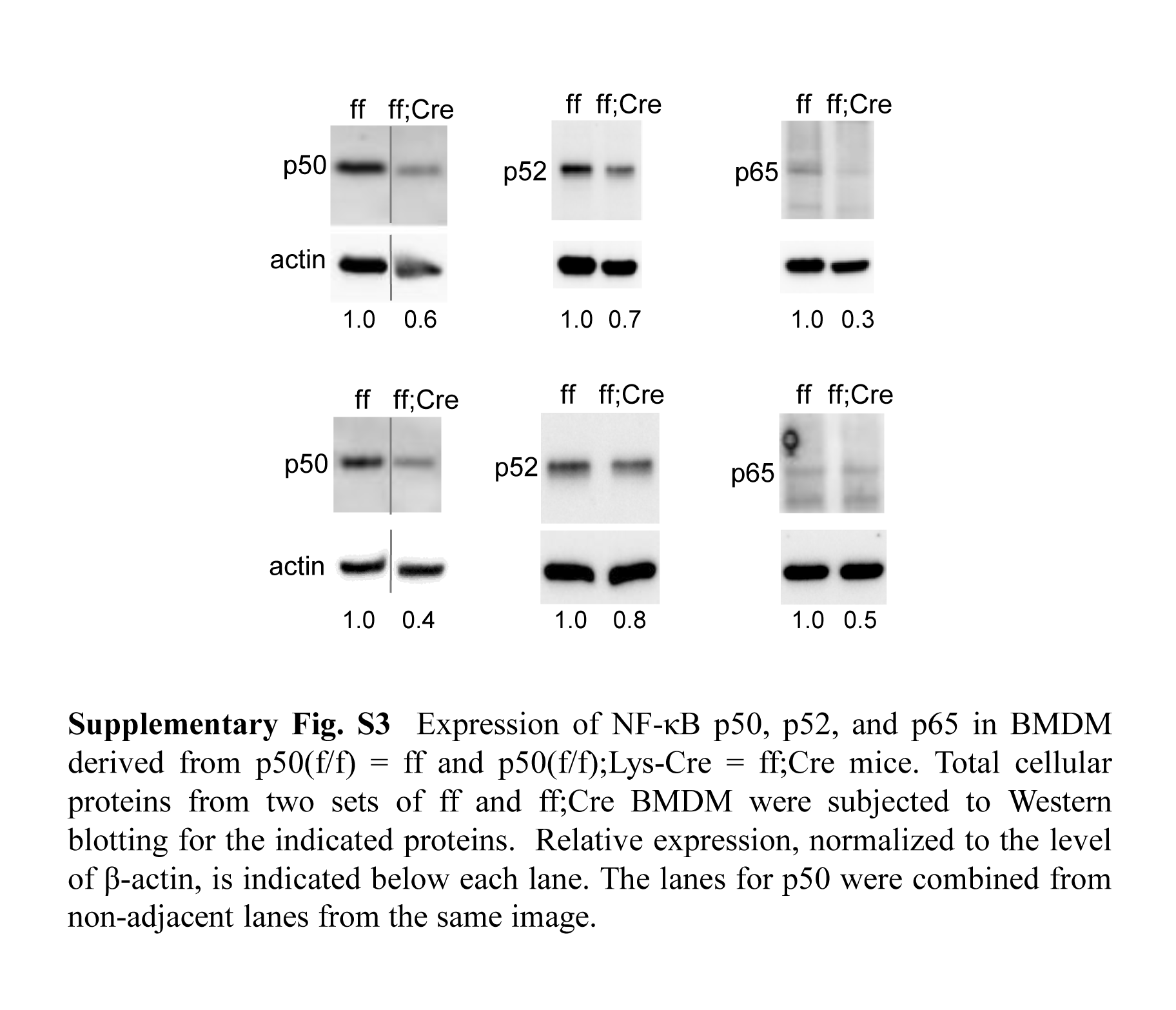

Supplement: Supplementary file 3 — Fig. S3. Expression of NF‐κB p50, p52, and p65 in BMDM derived from p50(f/f) = ff and p50(f/f);Lys‐Cre = ff;Cre mice. [file MOL2-15-1783-s001.tif]

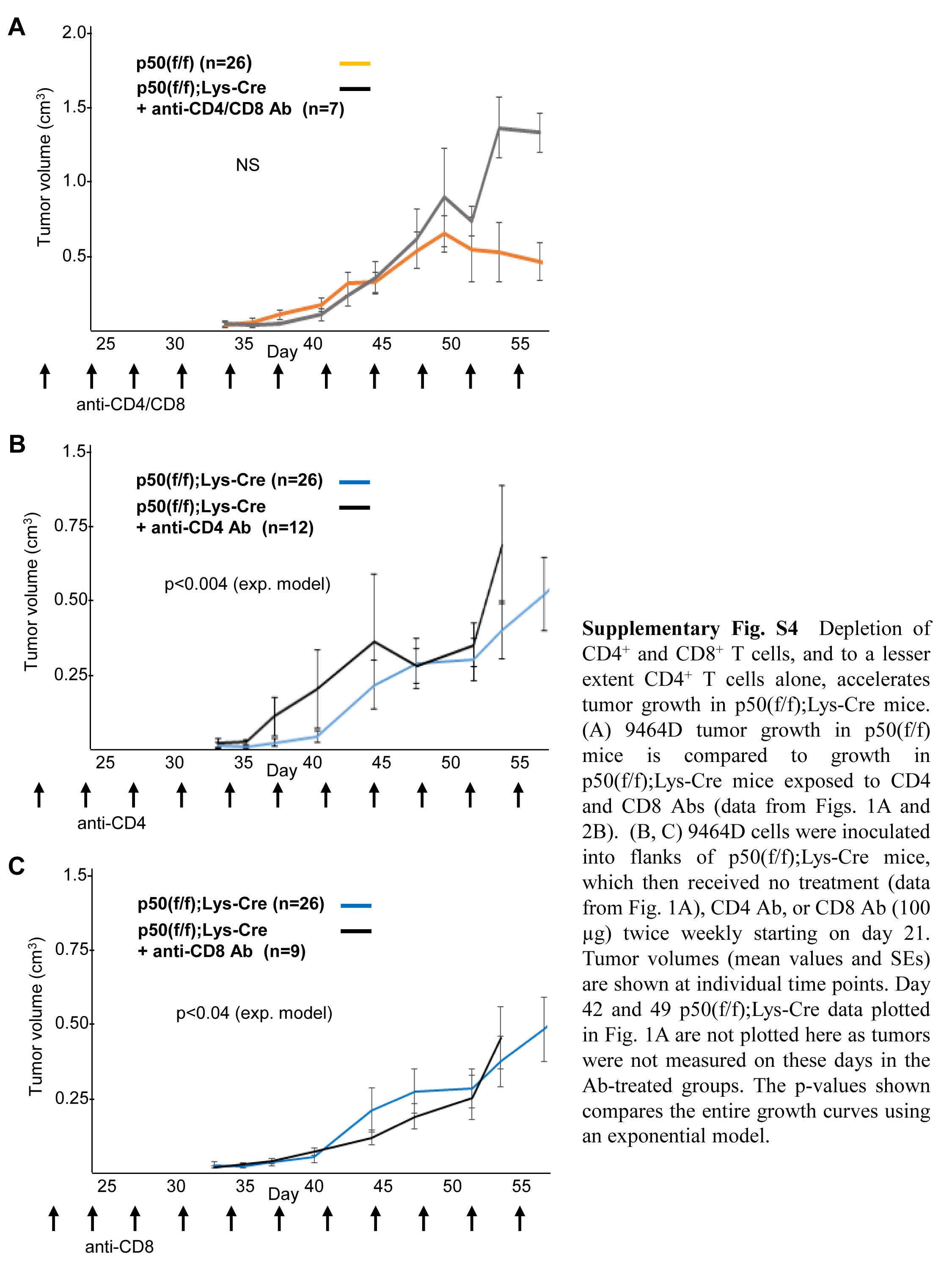

Supplement: Supplementary file 4 — Fig. S4. Depletion of CD4+ and CD8+ T cells, and to a lesser extent CD4+ T cells alone, accelerates tumor growth in p50(f/f);Lys‐Cre mice. [file MOL2-15-1783-s002.tif]

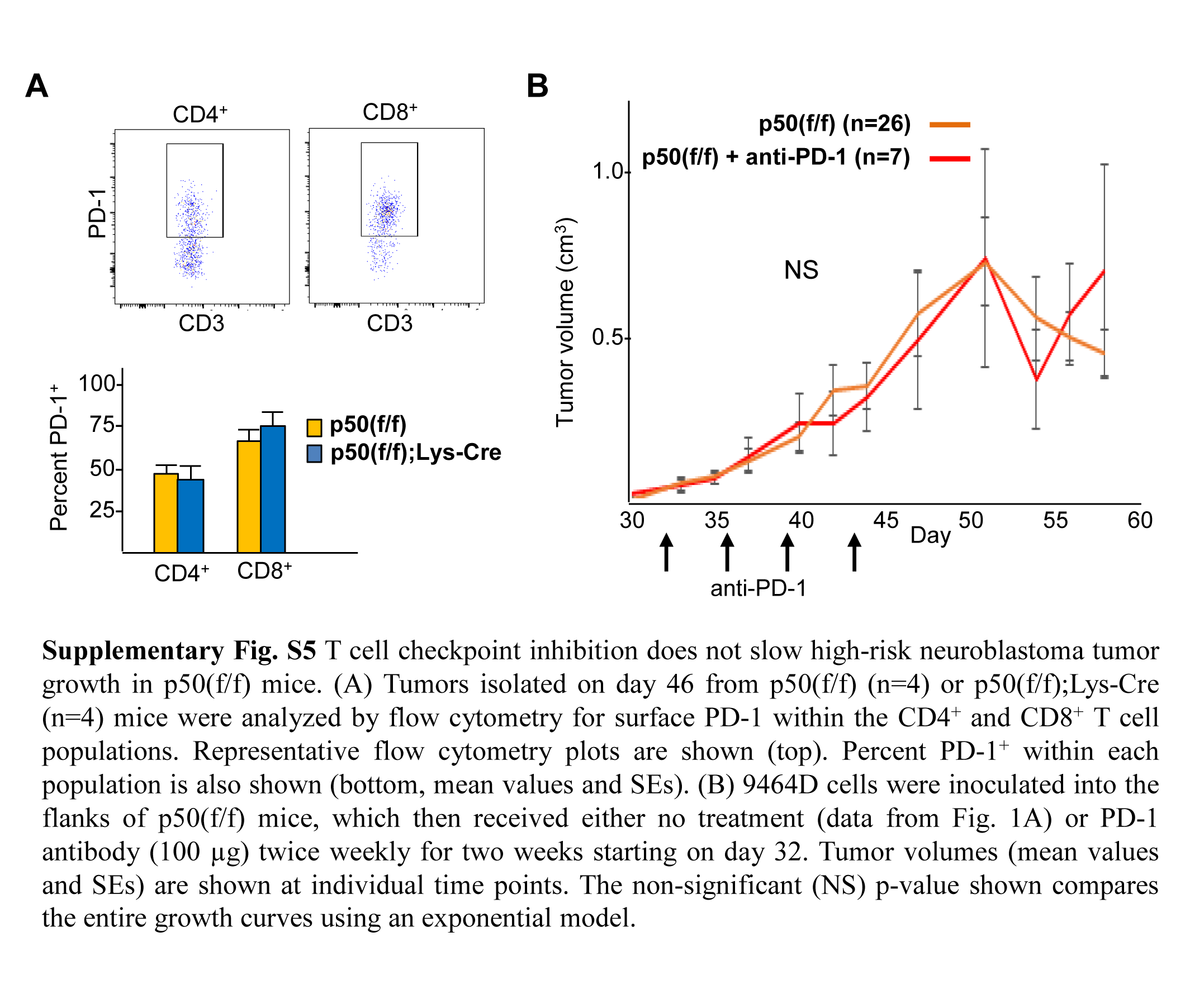

Supplement: Supplementary file 5 — Fig. S5. T cell checkpoint inhibition does not slow high‐risk neuroblastoma tumor growth in p50(f/f) mice. [file MOL2-15-1783-s006.tif]

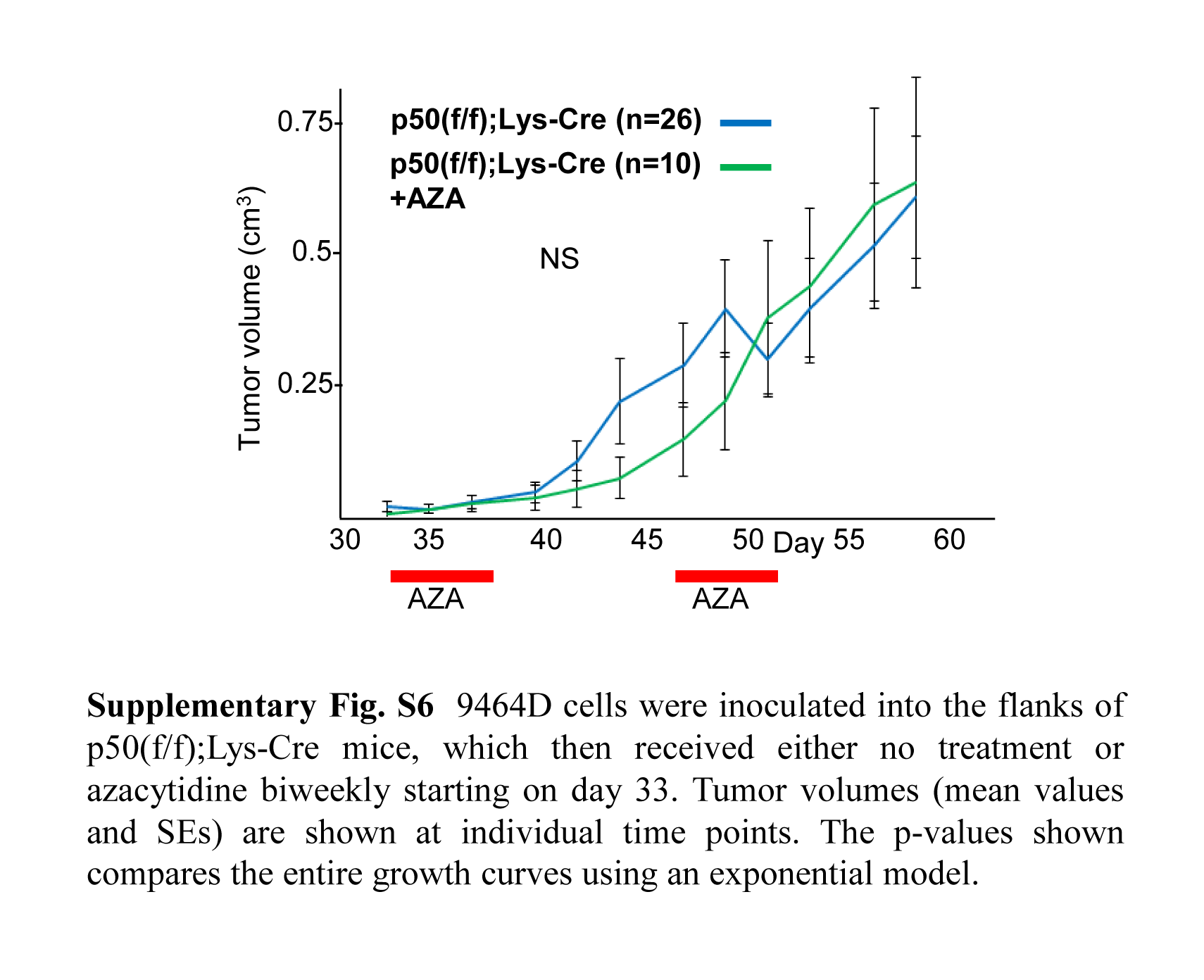

Supplement: Supplementary file 6 — Fig. S6. 9464D cells were inoculated into the flanks of p50(f/f);Lys‐Cre mice, which then received either no treatment or azacytidine biweekly starting on day 33. [file MOL2-15-1783-s003.tif]

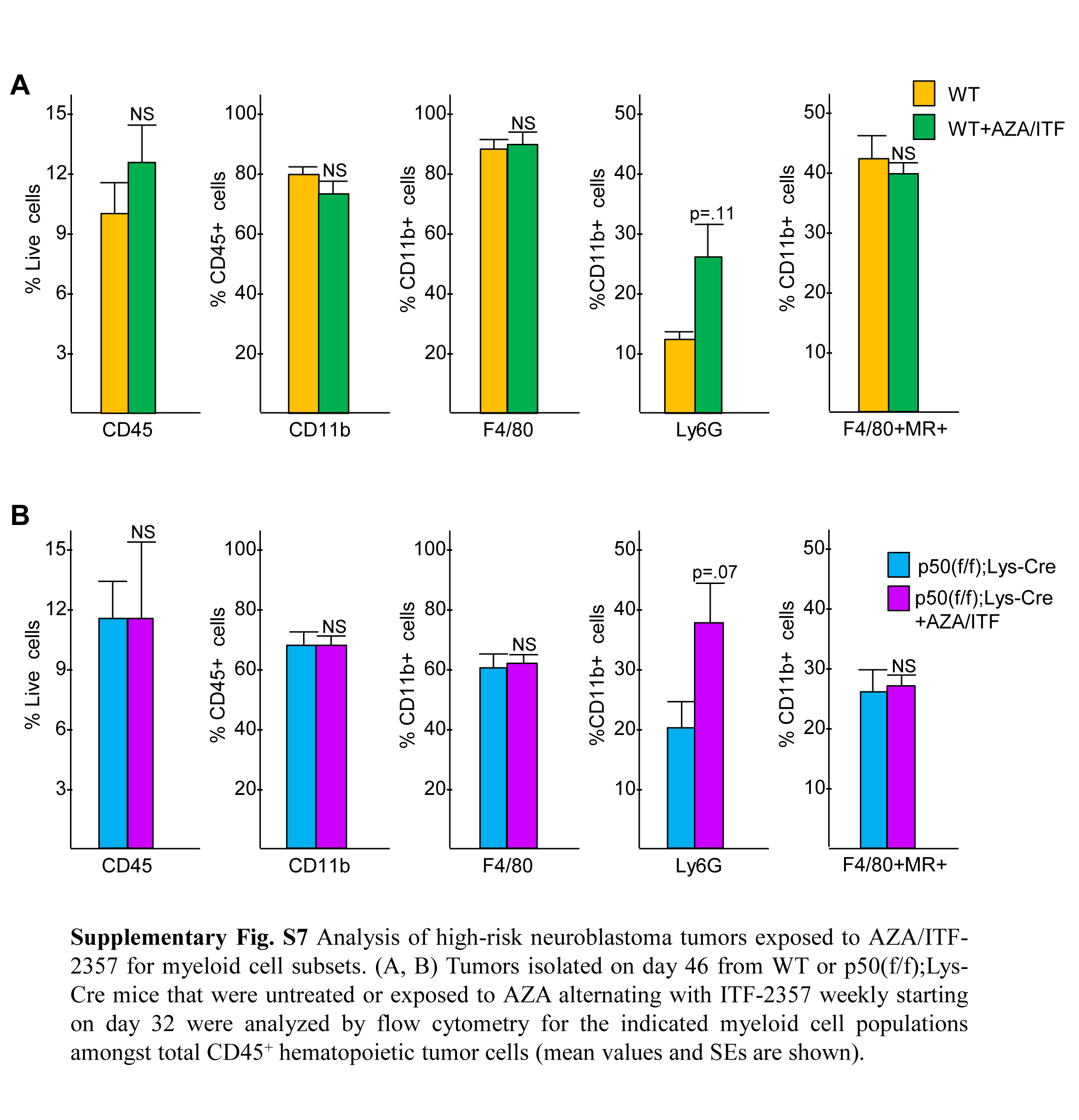

Supplement: Supplementary file 7 — Fig. S7. Analysis of high‐risk neuroblastoma tumors exposed to AZA/ITF‐2357 for myeloid cell subsets. [file MOL2-15-1783-s005.tif]

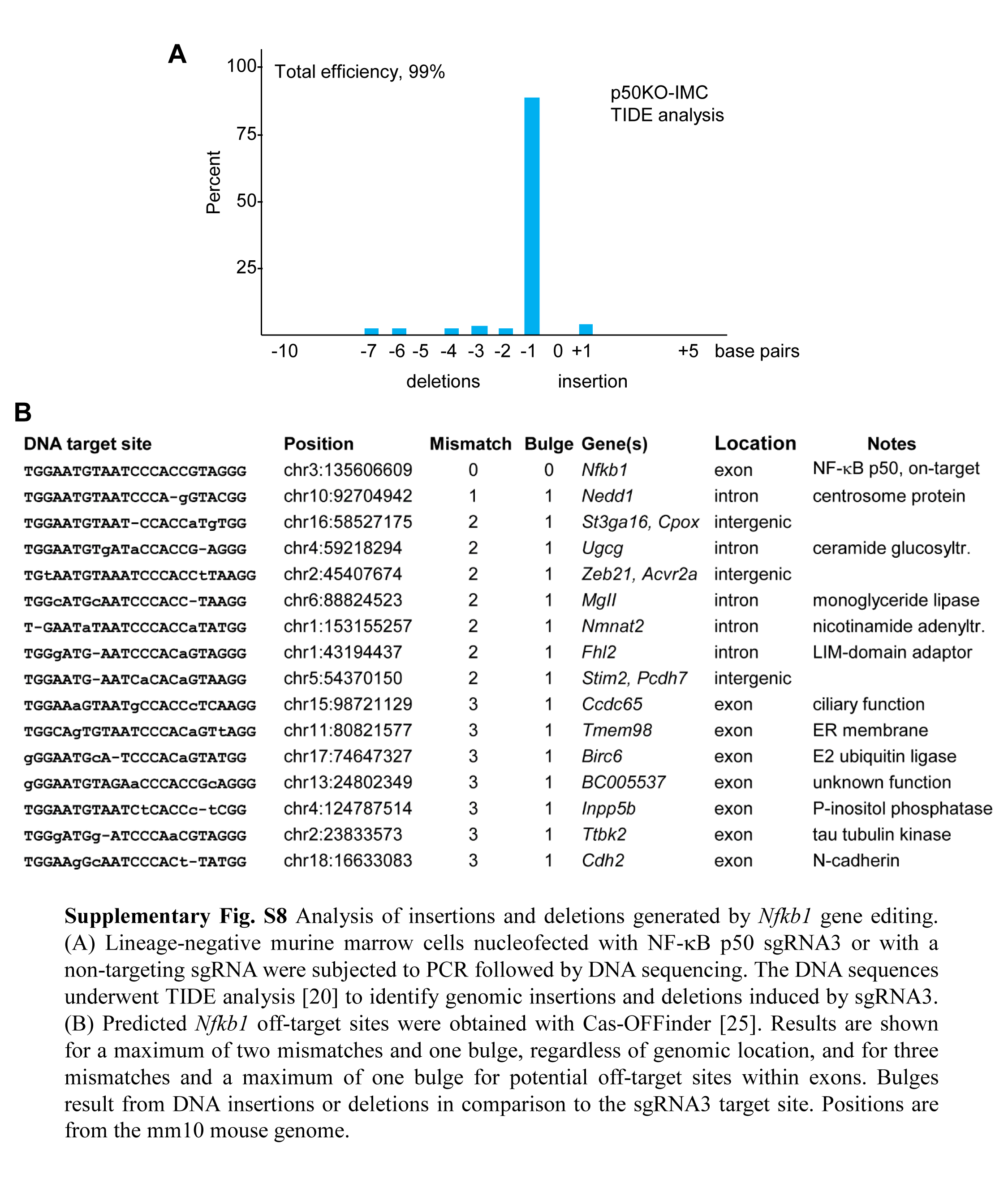

Supplement: Supplementary file 8 — Fig. S8. Analysis of insertions and deletions generated by Nfkb1 gene editing. [file MOL2-15-1783-s008.tif]
